# Colony morphotype variation in *Burkholderia:* implications for success of applications and therapeutics

**DOI:** 10.1128/jb.00521-24

**Published:** 2025-04-14

**Authors:** Pauline M. L. Coulon, Kirsty Agnoli, Garry S. A. Myers

**Affiliations:** 1 Australian Institute for Microbiology and Infection, Faculty of Science, University of Technology Sydney 1994, Sydney, New South Wales, Australia; 2 Department of Plant and Microbial Biology, University of Zurich 30975, Zürich, Switzerland; University of Virginia School of Medicine, Charlottesville, Virginia, USA

**Keywords:** *Burkholderia*, phase variation, virulence factors, therapeutics, environmental benificial, opportunistic infections

## Abstract

The *Burkholderia* genus includes both environmental and pathogenic isolates known for their phenotypic plasticity and adaptability. *Burkholderia* spp. are intrinsically resistant to many antibiotics, often requiring prolonged therapies during infection. A key feature of *Burkholderia* spp. is colony morphotype variation (CMV), which allows for rapid adaptation to environmental changes and influences virulence, antibiotic resistance, and pathogenicity by impacting the expression of key virulence factors such as lipopolysaccharides, extracellular DNA, efflux pumps, and flagella. While alternative treatments, such as vaccines and phage therapies, hold promise, CMV has the potential to undermine their efficacy by modifying essential therapeutic targets. Despite its importance, the prevalence and underlying mechanisms of CMV remain poorly understood, leaving critical gaps in our knowledge that may hinder the development of sustainable solutions for managing *Burkholderia* infections. Addressing these gaps is crucial not only for improving infection management but also for enabling the safe reuse of *Burkholderia* in biotechnology, where their plant growth-promoting and bioremediation properties are highly valuable. Our goal is to raise awareness within the scientific community about the significance of CMV in *Burkholderia*, highlighting the urgent need to uncover the mechanisms driving CMV. A deeper understanding of CMV’s role in virulence and resistance is essential to developing robust, long-term therapeutic strategies.

## CLINICAL RELEVANCE OF *BURKHOLDERIA*

**Fig 1:**
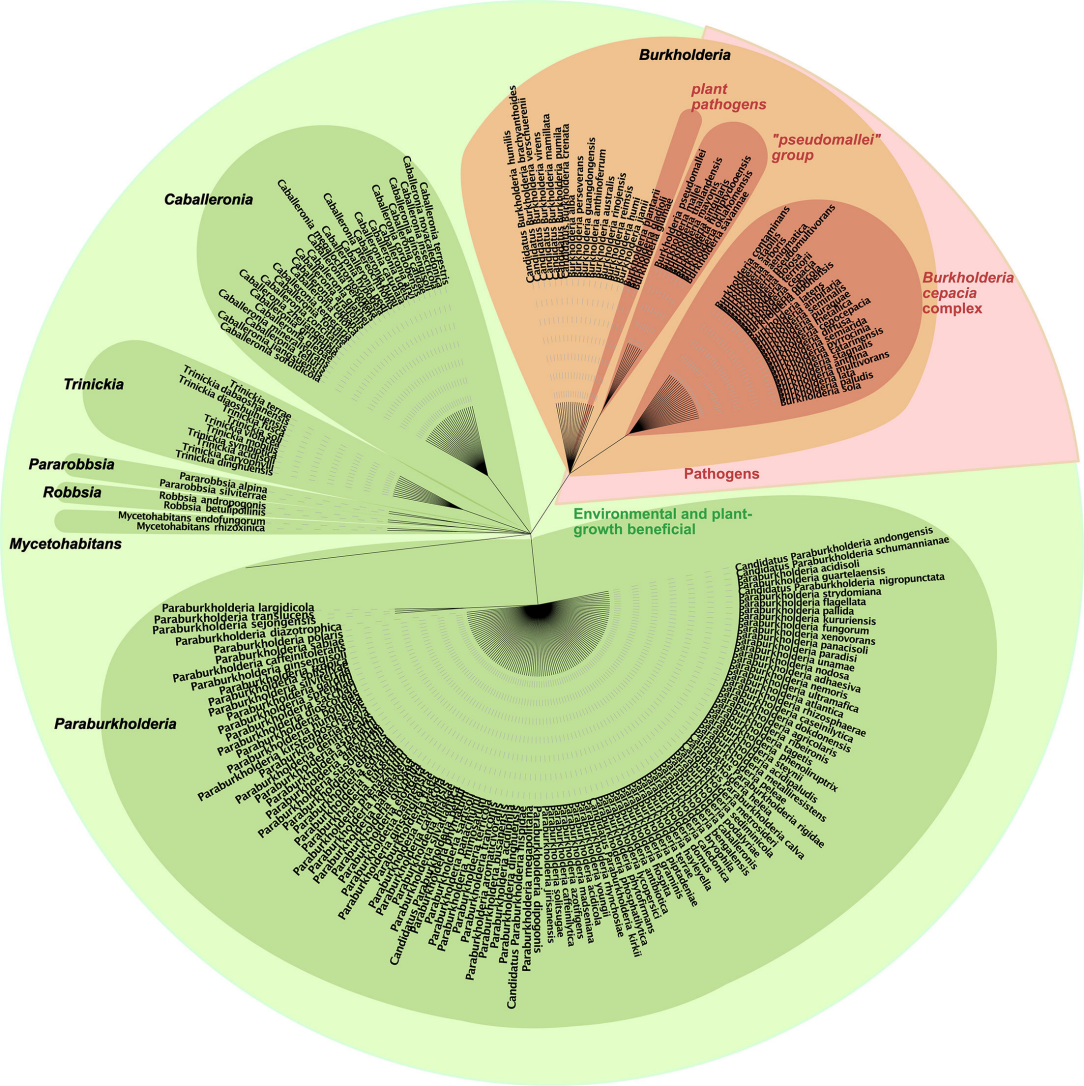
*Burkholderia* genus phylogeny based on NCBI taxonomy IDs. The tree was generated using PhyloT v.2 and iTOL v.6 (27). Figure updated from Eberl and Vandamme (17). Plant and environmental beneficial species are represented in green; opportunist pathogenic groups are represented in red. *Burkholderia* is represented in orange because some species can be both beneficial for the environment and pathogenic to specific populations.

The *Burkholderia* genus, comprising over 90 species (1, 2), is a member of the β-proteobacteria subphylum and is widely distributed in various environments such as the atmosphere, soil, water, plant rhizosphere, animals, and humans (3–7). The original *Burkholderia* genus has been separated into seven genera over the last decade (*Burkholderia*, *Paraburkholderia*, *Trinickia*, *Caballeronia*, *Mycetohabitans*, *Robbsia*, and *Pararobbsia*; Fig. 1), and the term *Burkholderia sensu lato* is now used to refer to these closely related genera collectively. The genus name *Burkholderia* has been retained for the clade containing (i) plant pathogens, including three species involved in rice disease (*Burkholderia plantarii*, *Burkholderia glumae*, and *Burkholderia gladioli*) (8–10); (ii) the *Burkholderia pseudomallei* complex (Bpc), comprising eight species, which includes *B. pseudomallei* and *B. mallei*, the causative agents of melioidosis and glanders in mammals and equines, respectively (11–16); and (iii) the *Burkholderia cepacia* complex (Bcc), a group of opportunistic human pathogens that includes at least 24 species (17–22), in which a few species are responsible for “*cepacia* syndrome” in immunocompromised patients, particularly those with cystic fibrosis (CF) (23–26).

Approximately a decade ago, the establishment of the *Paraburkholderia* clade was proposed to separate the plant-beneficial and environmental *Burkholderia* spp. from plant and mammalian pathogenic groups (2). This distinction was based on genomic guanine-cytosine content, sequence indels, the absence of known virulence factors (e.g., secretion systems), and the lack of virulence in the *Caenorhabditis* infection model (2, 28). However, as Eberl and Vandamme have emphasized, the absence of virulence in non-mammalian infection models does not necessarily equate to avirulence in mammals (17). For instance, many *Burkholderia multivorans* strains—one of the most prevalent Bcc species infecting immunocompromised patients and those with cystic fibrosis (29, 30)—are not virulent in non-mammalian models (31, 32) despite producing most of the virulence factors considered essential for pathogenicity (28, 33–35). Therefore, members of the *Paraburkholderia* clade require further phenotypic characterization, and clinical outcomes cannot be accurately predicted by model-based virulence studies alone. Indeed, although there are few reports of the infection of humans by *Paraburkholderia* spp., some reports do exist (36–38).

While *Paraburkholderia* spp. are generally beneficial for plants (17, 39), some *Burkholderia* spp. are known phytopathogens. The most notable examples are *Burkholderia gladioli*, *Burkholderia glumae*, and *Burkholderia plantarii*, which cause gladiolus disease, rice panicle blight, and rice seedling blight, respectively, across Asia and North America (8, 9, 40, 41). Interestingly, while *B. glumae* and *B. plantarii* have not been found infecting humans (1, 42–44), *B. gladioli* is frequently identified in CF patients in the USA, as are the Bcc members *Burkholderia cenocepacia*, *Burkholderia multivorans*, and *Burkholderia vietnamiensis*, which are the three most prevalent Bcc species in CF infections (29).

In contrast, some Bcc species exhibit beneficial traits, such as bioremediation and plant growth promotion (e.g., *Burkholderia ambifaria* and *B. vietnamiensis*) (45–49), while others are plant pathogens, like *Burkholderia cepacia*, which is pathogenic to onions (50). Due to their beneficial properties, Bcc species were used previously in biotechnology and agriculture (51), but their role as opportunistic pathogens, especially among immunocompromised patients—such as those with CF (30, 52–54), chronic granulomatous disease (55, 56), and cancer (57), as well as vulnerable populations, including the elderly and infants (58, 59)—has led to the withdrawal of these products from the market (60, 61), followed by the release of a new use rule for Bcc by the Environmental Protection Agency (https://www.federalregister.gov/documents/2003/06/13/03-15010/burkholderia-cepacia-complex-significant-new-use-rule). Bcc infections mostly occur through direct environmental contact (62), contamination of surfaces or pharmaceutical products (63–66), and patient-to-patient transmission via aerosolized particles or direct physical interaction (67–71). In CF patients, the combination of thick mucus production and the ability of Bcc to thrive in stressful conditions (71, 72) results in infections ranging in severity from asymptomatic carriage through a decline in pulmonary function to fatal lung deterioration known as *cepacia* syndrome (73–75). Even following lung transplantation, CF patients infected with Bcc remain at risk of bacterial pneumonia and lung abscesses (48, 76–78).

In recent years, infections of CF patients with *B. pseudomallei* have been reported (79–82). *B. pseudomallei* is a pathogen endemic to Asia, Africa, Central and South America and Northern Australia (83) – but is not classified as a neglected tropical disease yet. Recently, in the USA, three melioidosis cases were reported, which were attributed to local environmental exposure rather than acquisition abroad (84). This suggests that *B. pseudomallei* is naturally present in the US environment.

In less severe cases, *B. pseudomallei* causes contained skin abscesses, but in severe cases, it can rapidly lead to sepsis with bacteremia, affecting organs such as the lungs (50% of cases), spleen, prostate, and brain (4%) (85, 86). The presentation of the disease is dependent on the infection route; percutaneous infection tends to result in skin abscesses, but infection via inhalation, which often occurs due to aerosolization during severe tropical storms, favors the more severe pneumonic presentation of disease, which more frequently leads to fatal fulminant sepsis (87, 88). As climate change increases the frequency of heavy rains and floods, as well as cyclones, it is expected that the occurrence of severe *B. pseudomallei* infections will also increase and could spread to new areas (89).

Limmathurotsakul and colleagues (90) estimated an annual incidence of 169,000 melioidosis cases, with a mortality rate of 52%. While most melioidosis cases (85%) occur acutely within 1–21 days after exposure, about 10% develop chronically, particularly in immunocompromised patients, those on corticosteroids or immunosuppressive therapy, and those with diabetes, chronic kidney disease, chronic lung disease, or alcoholism (83, 91, 92).

*Burkholderia pseudomallei* infections have been reported in various animals, including goats, sheep, exotic animals, and pets, leading to ulcer formation in multiple organs and symptoms similar to those observed in humans (93). The predominantly equine disease glanders, which is caused by *B. mallei*, is often symptomatically similar to melioidosis, resulting in pulmonary abscesses (11–13, 94). It has been established that *B. mallei* is a host-adapted derivative of *B. pseudomallei* that has undergone extensive genome reduction (95, 96). As a result, *B. mallei* is non-motile and has an obligate intracellular lifestyle in mammals. Infections in humans are rare, but when they do occur, they are frequently fatal (12, 97). Other members of Bpc are *Burkholderia oklahomensis*, *Burkholderia singularis*, and *Burkholderia thailandensis*, which are opportunistic pathogens, and *Burkholderia mayonis*, *Burkholderia humptydooensis*, and *Burkholderia savannae*, which have not yet been associated with disease (88, 98–100).

*Burkholderia* spp. exhibit extensive phenotypic plasticity, including CMV, which allows them to inhabit diverse niches, from plant roots to the CF lung (101). This adaptability also enables *Burkholderia* to persist in water, raising concerns about transmission to vulnerable populations via contaminated aqueous pharmaceuticals (e.g., saline solutions containing benzalkonium chloride) (102–104). The basis for this adaptability lies in their large genomes (~7 Mbp), which are organized into multiple replicons (typically two or three chromosomes with plasmids). Additionally, *Burkholderia* spp. regulate the production of secondary metabolites, including antimicrobial compounds and enzymes capable of degrading environmental substances, further enhancing their survival and versatility (3, 105, 106).

## *BURKHOLDERIA* CMV PREVALENCE DATA ARE BIASED BY THE CURRENT COMMUNITY FOCUSES

**Fig 2:**
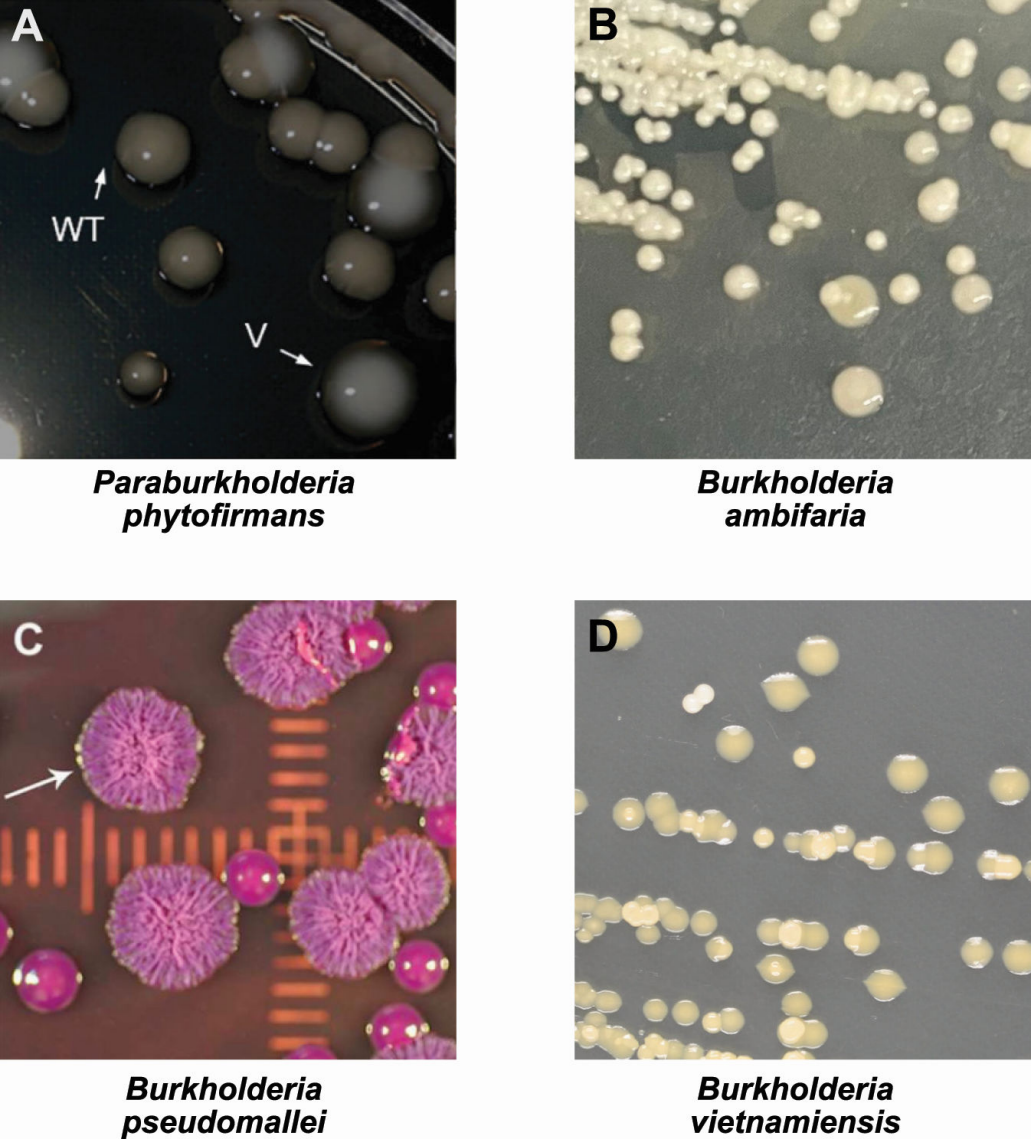
Colony morphotype variation (A) *P. phytofirmans* PsJN, yellow parental colonies (WT) and white variants (V) modified from Rondeau and colleagues (112); copyright *ASM* journal, order license ID 1533523–1. (B) *B. ambifaria* rough parental colonies with small smooth variants. (C) *B. pseudomallei,* rough parental colonies with small smooth variants, modified from Shea and colleagues (114). (D) *B. vietnamiensis* big yellow smooth parental colonies with small yellow and white smooth variants. WT, wild type.

Over the years, CMV has been reported among *Burkholderia*, in isolates taken from either the same time point or over the course of a mammalian infection (e.g., human [107, 108] and pig [109]) or when cultured in the laboratory (110–113). CMVs are distinct colony types that arise from a parental isolate due to genomic variation, difference in expression of targeted genes, or epigenetic modulation (112, 114–121). For example, the plant-associated species *Paraburkholderia phytofirmans* exhibits two CMVs when cultured under static conditions (112). This indicates that even plant-associated symbionts require CMV to better respond to environmental changes. Similarly, opportunistic clinical isolates of *B. ambifaria* revert to environmental-like CMVs when grown on rich medium. These CMVs show a reduction in virulence factors relevant to host invasion and an increase in competitive properties such as a stronger β-galactosidase activity allowing the hydrolysis of cellobiose; an ability to metabolize saccharose, xylose, and polyols, mainly found in plants and rhizosphere; and an increased production of extracellular polysaccharide (EPS) involved in the attachment of bacteria to the roots. These characteristics facilitate plant colonization, meaning that they are better adapted to the rhizosphere (113). *B. pseudomallei* forms up to seven CMVs under stress conditions mimicking infection, such as prolonged stationary phase, starvation media, presence of antibiotics, and osmotic and oxidative stresses. Most of these morphotypes revert to the wild-type CMV once the stress is removed, showing that environmental *B. pseudomallei* uses reversible mechanisms to adapt during host invasion, where virulence factors are no longer required in the absence of environmental pressures (110, 111, 122) (Fig. 2).

Of the nearly 12,200 *Burkholderia* isolates registered in PubMLST (123), only 92 isolates (0.07%) have been described to undergo CMV (Table 1). Among these, 76 isolates and their respective CMVs varied in their production of virulence factors, with some also differing in virulence. The research community focuses on genomic plasticity and infection surveillance, rather than on phenotypic variation, likely explaining why CMV appears to be a rare phenomenon in *Burkholderia*. However, CMV is a common bacterial strategy for rapidly adapting to new environments, suggesting that its occurrence is underreported in the literature.

**TABLE 1:** Impact of CMV on phenotypes among *Burkholderia* isolates^*a*^

Species	Strain	Origin	No. of CMVs	Phenotypes	Comments	Reference
*B. ambifaria*	AU0212	CF isolate (USA)	2	EPS and hemolysin production, antifungal and cholesterol oxidase activities, and virulence	Loss of pC3	(113, 124)
	AU4157	Clinical isolate	2			
	AU8235	Clinical isolate	2			
	CEP0516	CF isolate (Australia)	2			
	CEP0617	Clinical isolate	2			
	CEP0958	CF isolate (Australia)	2			
	CEP0996	CF isolate (Australia)	2	EPS, flagellum, hemolysin, siderophore, antimicrobial, protease, cholesterol oxidase, and virulence		
	HSJ1	CF isolate (Canada)	2	EPS, hemolysin, antifungal, cholesterol oxidase, biofilm, macrophage infection, and virulence	Quorum sensing and DNA methylation involved in the emergence of phase variants	
*B. cenocepacia*	K56-2	CF isolate (Canada)	2	Antimicrobial activity, EPS, protease, substrate utilization, biofilm, quorum sensing, and virulence	Mutation in *shvR*	(116, 125)
	IST439/IST4103/IST4129/IST4130/IST4131/IST4134	CF isolate (Portugal)	3	Biofilm, EPS, cell size, and antibiotic resistance	Rough, semi-rough, and smooth colonies isolated from the same patient belonging to two ST lineages	(52, 108, 126)
	IST4110/IST4112/IST4113		3	Biofilm, EPS, cell size, antibiotic resistance, and hydrophobicity		(52, 108)
	IST4116A/IST4116B		2	Biofilm, EPS, cell size, antibiotic resistance, and hydrophobicity	Rough, semi-rough, isolated at the same time from the same patient	
	Bcc060/Bcc061	CF isolate (Canada)	2	Biofilm and mucoidy		(107)
	Bcc002/Bcc003		2			
	Bcc043-Bcc046		4	Biofilm and swimming		
	Bcc049/Bcc050		2			
	Bcc063/Bcc064		2			
	Bcc096/Bcc097		2			
	Bcc118/Bcc119		2			
	Bcc121/Bcc122		2			
	Bcc158/Bcc159		2			
	Bcc205/Bcc206		2			
	Bcc065-Bcc068		4	Biofilm, swimming, and virulence		
	Bcc069/Bcc070		2			
	Bcc187-Bcc190		4			
	Bcc192/Bcc193		2			
	Bcc212/Bcc213		2			
	Bcc214/Bcc215		2			
	Bcc015/Bcc016		2			
	Bcc115		1	Biofilm, swimming, mucoidy, and virulence	First isolate sampling in the patient, followed by isolates carrying pC3	
	Bcc020/Bcc021		2	Swimming		
	Bcc027/Bcc028		2			
	Bcc029/Bcc030		2			
	Bcc125/Bcc126		2			
	Bcc160/Bcc161		2			
	Bcc163/Bcc164		2			
	Bcc208/Bcc209		2			
	Bcc008/Bcc009		2	Swimming and mucoidy		
	Bcc013/Bcc014		2			
	Bcc073/Bcc074		2			
	Bcc084/Bcc085		2			
	Bcc112/Bcc113		2			
	Bcc071/Bcc072		2	Virulence		
	Bcc075/Bcc076		2			
	Bcc220/Bcc221		2			
	Bcc129/Bcc130		2		Suspected chromosome fusion	
*Burkholderia contaminans*	MF16_B/MF17_B	CF isolate (Argentina)	2	Antifungal, hemolysis, protease, and swimming		(127)
	i_S/L_S		2	Protease and swimming		
	466_S/467_S		2			
*B. mallei*	ATCC 23344	Melioidosis isolate (Burma)	3	Antimicrobial resistance, LPS, macrophage infection, and murine virulence		(128)
*B. multivorans*	BM11 (or D2095)/BM11L	CF isolate (Canada)	2	Antibiotic sensitivity, motility, adhesion, biofilm, osmotic stress tolerance, virulence, and mucoidy	Isolated from the same sample, mutation in acetyl CoA enzyme	(129)
	BM11/BM11-nmv9/BM11-nmv9r		3		Obtained under stress conditions, mutation in OmpR	
	BM11L/BM11L-nmv1/BM11L-nmv2		3			
	BM6/BM6-nmv1		2			
	BM7/BM7-nmv1		2			
	C1394	CF isolate (England)	2	EPS and pili		(130)
*B. pseudomallei*	UMC074	Collection strain (Malaysia)	2	Adherence, invasion, and plaque forming on human epithelial cells		(131, 132)
	CTH	Clinical isolate (Malaysia)	2	Biofilm, virulence in *Caenorhabditis elegans*		(133)
	OCY		2			
	TOM		2	Acyl-homoserine lactone, biofilm, and virulence in *Caenorhabditis elegans*		
	VL		2			
	NCTC 10274	Clinical isolate (Malaysia)	2	Antibiotic resistance		(111)
	NCTC 7431	Clinical isolate (Unknown)	2			
	CB/CS	Melioidosis isolate (Malaysia)	2	Antibiotic and pH sensitivity		(134)
	OB/OS		2			
	MSHR5848	Melioidosis isolate (USA)	2	Cell morphology, biochemical sensitivity or utilization, macrophage survival and activity, and virulence	Indel in the promoter of potential lipoprotein chr2 cluster of bacteriophage-associated genes on chromosome 2 upregulated in S phenotype	(114, 135)
	K96243	Melioidosis isolate (Thailand)	6	Antimicrobial resistance, eDNA, LPS, macrophage infection, murine persistence and virulence, and colony color (YA, YB, and white)	YA and YB obtained under hypoxic conditions. YelR regulator responsible for phenotype, no mutations detected in the yellow variants	(115, 128, 136)
	1	Clinical isolate (Thailand)	2			(109)
	2		2			
	3		2			
	4		2			
	5	Pig isolate (Thailand)	2			
	153	Melioidosis isolate (Malaysia)	3	Increased expression of flagellin and arginine deiminase system components which facilitate acid tolerance		(122)
	1026b	Melioidosis isolate (Thailand)				(128)
	E8	Soil (Thailand)	8			(137)
	DT	Melioidosis isolate (Taiwan)	7			
	NTC13392		8		Obtained after passage in mice, no significant genomic difference between morphotypes	(118)
	164	Melioidosis isolate (Thailand)	3	Biofilm, protease, lipase, elastase, motility, adherence, replication in macrophages and epithelial cells, and virulence in mice		(110, 136)
	153		3			
	4095	Clinical isolate (Thailand)	2	Internalization, OPS, and mucoidy	No mutation and difference in expression of wibA	(138)
	10457A		2			
	10971B		2			
	11017A		2			
	MSHR295	Soil (Australia)	2			
	B3	Soil (Thailand)	3	Invasion and antimicrobial peptide resistance		(136)
	B4		3			
*B. thailandensis*	BtE264		2	Biofilm	IS-mediated RecAdependent duplication of a 208.6 kb region: flat (1) rough (2) smooth (3)	(139)
*Burkholderia ubonensis*	MSMB2035/MSMB2036	Soil (Australia)	2		Loss of pC3	(140)
*P. phytofirmans*	PsJN	Plant-associated bacterium	2		Flat yellow to white raised CMV; mutation of *hscA* and *iscS* genes belonging to iron-sulfur cluster but does not explain all phenotypic differences between CMV	(112)

^*a*^ CF, cystic fibrosis; eDNA, extracellular DNA; LPS, lipopolysaccharide; OPS, O-antigen lipopolysaccharide.

Screening for phenotypes linked to virulence factors offers a cost-effective, high-throughput approach to begin to understand how CMV influences virulence and pathogenicity in infection models. However, exploring the underlying mechanisms of CMV requires more resources than simple phenotypic assays. To date, six *Burkholderia* isolates have been linked to identified mechanisms, including mutations in global regulators (116, 117, 141–143), two-component systems (129), genome reduction and duplication (79, 125, 139, 140, 144–146), bacteriophage cluster integration (147), and DNA methylation (148).

CMV can be an integral part of host colonization. For example, a study using *B. pseudomallei* K96243 investigated the occurrence of white colonies (more frequently isolated from the environment) and two yellow colony variants (YA and YB) that showed increased resistance to hypoxic stress (consistent with the conditions encountered upon entering a host organism) (115). It was found that upregulation of *yelR*, which encodes a σ-54-dependent regulator, gave an identical morphotype to YB. YB showed attenuated virulence in a murine model but increased resistance to hypoxic stress. Interestingly, it was found that only the YB morphotype was able to colonize and persist in the harsh conditions of the murine stomach. Due to the reversible nature of these variations, the YB phenotype could later revert to the parental phenotype, with a concomitant increase in virulence. It should be noted that, while the white colony form is most commonly isolated from the environment, clinical isolates are much more variable, supporting the importance of CMV for *B. pseudomallei* infection (115). The authors found that the development of yellow variants was probably stochastic, with these variants becoming more prevalent under hypoxia due to their selective advantage, rather than *yelR* expression being controlled by oxygen levels (115). *B. pseudomallei* is known for producing a range of colony morphotypes, with seven general morphotypes reported in the literature (110). These variants also show differences in their expression of virulence determinants. A general increase in antibiotic resistance and virulence demonstrated by small-colony variants (SCVs) has been reported (111, 149). Of the seven major *B. pseudomallei* morphotypes, rough colony variants have been reported to predominate in clinical melioidosis isolates, making up 83.8% of 212 clinical isolates from patients with melioidosis tested in a study by Chantratita and colleagues (110). A study by Tandhavanant and colleagues investigated two CMVs, that they classified as type II (small, rough) and type III (large, smooth) compared to the common, parental “cornflower head” morphotype (136). These morphotypes developed for a selection of clinical isolates under nutrient limitation. The phenotypes displayed by these morphotypes (e.g., persistence in cell lines) were inconsistent, probably due to differences in genetic content in the different strains. This study shows the importance of determining the relationship between genomic content and virulence in *B. pseudomallei* strains, while also taking into account the CMVs present (115).

In the plant-beneficial bacterium *P. phytofirmans*, mucoid colony variants were isolated from pellicles that showed more robust biofilm formation on plant roots compared to the parental isolate (101). These variants showed a loss of motility and an increase in EPS production and GroEL chaperonin expression. These phenotypic changes resulted from mutations in two genes involved in the iron-sulfur complex. The *Escherichia coli* homologs of these genes are known to be important for the maturation of a large number of iron-sulfur cluster proteins, which are essential for many housekeeping processes, such as respiration and DNA replication (112, 150). Duplication or genome reduction also aids bacterial adaptation in Bpc (139, 140, 146). For example, the RecA-dependent duplication of a 208.6 kb region in *B. thailandensis* E264 is responsible for the emergence of three CMVs (flat, rough raised, or smooth raised colonies), which favored biofilm formation over planktonic growth (139).

## CMV IN *B. MULTIVORANS* DEMONSTRATES THE IMPACT OF OMPR ON VIRULENCE

In a study by Silva and colleagues, 20 mucoid *B. multivorans* isolates from a CF patient sequentially sampled over 20 years were exposed to prolonged stationary phase (21 days) at 42°C (129). Following this, between 10% and 60% of colonies plated, depending on the culture, were small-colony variants, with most showing reduced mucoidy. Some of these SCVs were unable to produce EPS under the conditions tested and were designated non-mucoid variants (NMVs). Among the 15 NMVs isolated, 14 had at least one mutation in the *ompR* gene, part of the OmpR/EnvZ two-component system that regulates genes encoding outer membrane proteins (OMPs). One NMV had a 10 bp deletion in *bceF*, a gene within the cepacian EPS cluster controlled by OmpR. Loss of functional OmpR led to positive effects on CF lung epithelial adhesion, biofilm formation under high osmolarity, and motility. However, it had negative impacts on antibiotic sensitivity, osmotic stress tolerance, and virulence. Interestingly, in one NMV, partial OmpR function was restored by a reversion event, where a non-synonymous mutation (D13V) in *ompR* was acquired when cultured in salt medium supplemented with mannitol.

## CMV AND THE THIRD REPLICON OF THE BCC

Among the *Burkholderia*, Bcc shows CMV due to the variability and instability of its third replicon, pC3. This megaplasmid was originally designated chromosome 3 due to its large size (larger than 1 Mb in some strains), and the rRNA and tRNA genes encoded on it. However, this replicon, which shows high variation even between strains of the same species, is in fact non-essential and can be lost in its entirety. This generally leads to a reduction in phenotypes such as EPS production, antifungal activity, and virulence (125).

Although pC3 loss was first observed *in vitro*, some Bcc strains that lack pC3 have been reported. For example, Lee and colleagues carried out genomic analysis of serial isolates of *B. cenocepacia* from CF patients (107). They found many isolates that had undergone genome reduction, including two that had completely lost pC3 and showed the typical reductions in virulence (in a *Galleria mellonella* model) and mucoidy associated with pC3 loss. Furthermore, Wallner and colleagues conducted an analysis of the genomes of 31 different Bcc clinical and environmental isolates and found one environmental isolate, now classified as *Burkholderia orbicola* FL-5-3-30-S1-D7, from which pC3 was absent (151).

Approximately 50% of Bcc species have the *a*nti*f*ungal *c*ompound (*afc*) cluster, which is encoded on pC3 (141). This cluster and its regulator, shiny colony variant regulator (ShvR), were found to play an important role in CMV and virulence in *B. cenocepacia* K56-2 when spontaneous shiny variants were investigated for their virulence and other phenotypes (142, 143, 152). The disruption of *afc* genes or *shvR* in *B. cenocepacia* K56-2 results in reductions in antifungal activity, extracellular matrix production, and virulence. These are major phenotypes observed after pC3 loss (125). This suggests that for many Bcc members, the observed CMV and reduction in virulence after loss of pC3 is at least in part due to the loss of the pC3-encoded *afc* cluster and its regulator, ShvR.

In the Bcc, pC3 derives from a common ancestor replicon and has undergone rearrangements, leading to substantial variation in gene content (153, 154). There is no evidence of horizontal transfer of pC3 (140), but an intriguing question remains: could a pC3-null Bcc isolate potentially reacquire a pC3, either fully or partially, to revert to a more virulent and stress-resistant form?

## CMV IMPACTS O-ANTIGENS IN BOTH BCC AND BPC

Variation in LPS is common in Bpc and Bcc. CMVs have been observed in *B. pseudomallei* and *B. mallei* during long-term infections in mice, and naturally derived SCVs of *B. pseudomallei* exhibit increased expression of LPS biosynthesis cluster genes (128, 131). Wikraiphat and colleagues observed that approximately 10% of over 450 *B. pseudomallei* isolates produced mixed mucoid and non-mucoid populations on blood agar (138). The mucoid phenotype was associated with differences in O-antigen LPS (OPS) production, but these differences were not attributed to mutations or altered expression of the O-antigen acetylase *wbiA*. While mutations in other genes belonging to the LPS biosynthesis cluster cannot be excluded, other mechanisms, such as phase variation, could explain these phenotypes (113, 124).

In Bcc species, OPS production is modulated via *de novo* mutations, and this modulation is advantageous during longterm infection. While OPS is required for antibiotic resistance and macrophage invasion, its loss provides a significant benefit in evading host immune system responses and facilitates persistence in chronic infections (155, 156). However, the modulation of OPS seems to be species dependent. For instance, *B. cepacia* and *Burkholderia contaminans* tend to retain O-antigen, whereas the more prevalent species in CF, *B. cenocepacia* and *B. multivorans*, often become O-antigen negative during long-term infections (126, 155, 156). One study found that an ancestral, OPS-negative *Burkholderia dolosa* strain had undergone adaptation by regaining O-antigen production (157). This was mediated by non-synonymous mutations that corrected a premature stop codon in the *wbaD* gene. This occurred independently in nine infected individuals, highlighting the selective pressure for O-antigen presence after the establishment of colonization in the CF lung.

## RELEVANCE OF CMV IN PLANT-GROWTH PROMOTION AND ENVIRONMENTAL BENEFICIAL *PARABURKHOLDERIA* AND *BURKHOLDERIA* ISOLATES

The plant symbiont *P. phytofirmans* exhibits CMV, which enhances biofilm formation during *Arabidopsis* root colonization, without activating plant defense responses. This variation promotes a heterogeneous population, enhancing adaptability to diverse environmental conditions (112). In the case of the Bcc, CMV facilitates adaptation to fluctuating environmental conditions, for example, through the loss of plasmid pC3, a key factor in virulence (113, 124, 125). Although in some strains (e.g., *B. cenocepacia* K56-2) very efficient toxin-antitoxin systems are present which prevent the removal of pC3 without extensive genetic modification, the removal of pC3 can often be achieved in the laboratory by the introduction of a small plasmid bearing the single copy number pC3 origin of replication and markers for selection and subsequent counterselection (125, 144). This opens the doors for the construction of Bcc derivatives with reduced pathogenic potential, which could be used to enhance plant growth promotion or outcompete virulent Bcc strains, without concerns about pathogenicity. For example, Mullins and colleagues deleted pC3 from *B. ambifaria* Bcc0191, a plant-beneficial Bcc strain. The resultant strain retained its ability to protect against damping-off disease when applied to soil and was no longer able to persist within a murine respiratory infection model (158). These findings suggest that controlling CMV could enable the safe usage of *Burkholderia* for applications if pC3 reacquisition is found not to occur naturally.

## UNDERSTANDING THE UNDERLYING MECHANISMS OF CMV IS CRUCIAL FOR THERAPEUTIC DEVELOPMENT

*Burkholderia* spp. are intrinsically resistant to most commonly used antibiotics, often requiring up to 6 months of intensive antimicrobial therapy, which is not always successful (24, 25, 83, 107, 159). In the context of growing antibiotic resistance due to overconsumption and limited access to effective treatments against *Burkholderia*, the search for alternative therapeutics is more urgent than ever. The common mechanisms underlying antimicrobial resistance include (i) the acquisition of new resistance genes, (ii) alterations in drug targets, and (iii) variations in OMPs, as reviewed by Rhodes and Schweizer (160). Notably, CMV impacts some of these mechanisms, with modifications in OMPs, LPS (113, 128, 131, 132, 149), and efflux pumps (124) playing a significant role in antimicrobial resistance.

Vaccines and phage therapies present promising alternatives to combat antimicrobial resistance and could prove efficacious against *Burkholderia* infections. Outer membrane vesicles—used as a vaccine itself or a delivery method—as well as subunit vaccines represent a safer option compared to inactivated *Burkholderia* vaccines, especially for *B. pseudomallei*. Current vaccine candidates target conserved proteins such as those involved in type VI secretion systems, OMPs, flagella, and LPS, all of which play major roles in virulence (161–163).

Although Bcc remains a significant burden and is still lethal for patients with CF and those who are immunocompromised, the small size of the affected population makes it difficult for researchers to secure funding for vaccine development. Consequently, research has shifted toward alternative therapeutics, such as phage therapy (164). At least 34 phages have been identified with activity against *Bcc* (165, 166). However, in two separate incidences within the clinic, the use of phages as a last-resort therapy failed to clear Bcc infections, and both patients died (167, 168). This highlights the therapeutic limitations to clearing resistant infections. The potential for bacteria to modify or block phage receptors on their outer membranes or alter the production of EPS further complicates the efficacy of phage therapy (169).

All these new therapeutics are based on *Burkholderia* model strains and are far from being commercialized, and current research has not yet considered the challenges that CMV in *Burkholderia* presents. Isolates can exhibit variations in the same proteins that are targeted by vaccines and phages, as well as those involved in antibiotic resistance. For example, EPS is implicated in mucoidy, persistence, and colonization; extracellular DNA is involved in cell adherence and biofilm formation; flagella are essential for swimming motility; and LPS, efflux pumps, and porins are key players in antibiotic resistance and pathogenicity (79, 113, 122, 124, 125, 131, 132, 149) (Table 1). Consequently, the underlying mechanisms of CMV and its impact on protein production and modification must be considered to ensure optimal efficiency of therapeutics targeting these factors.

However, the relationship between CMV and phenotypic variation remains complex and unclear. For instance, when comparing two *B. pseudomallei* isolates producing morphologically identical CMVs, no consistent correlation was found between the CMV and typical phenotypes (110, 127). In contrast, a reduction in the production of several virulence factors—such as hemolytic activity, mucoidy, antimicrobial production, biofilm formation, and siderophore production—was observed across eight morphologically distinct *B. ambifaria* isolates (113, 124). Interestingly, six out of these eight CMVs had lost plasmid pC3, while the remaining two had undergone phase variation. This suggests that while CMV may influence *Burkholderia* pathogenicity and resistance, the broader impacts of CMV on phenotypic variation are still not well understood.

## PERSPECTIVES AND FUTURE DIRECTIONS

Our current understanding of CMV in *Burkholderia* remains limited, and this gap needs to be addressed. It is essential to design a comprehensive study aimed at determining the distribution and prevalence of CMV across both environmental and animal clinical *Burkholderia* isolates. Uncovering the underlying molecular mechanisms behind CMV will enhance our fundamental knowledge of evolution and behavior and our understanding of *Burkholderia* virulence, pathogenicity, and its response to treatment. The integration of these data into an accessible database would facilitate communication and collaboration, consistent with the One Health approach to increase efficiency in finding solutions. This knowledge could be translated and used in agriculture, environmental management, and biotechnology to generate safer *Burkholderia* biocontrol, and also in health to find biomarkers for diagnosis and to design novel and more effective therapeutics, contributing to long-term management of *Burkholderia* infections and combating antimicrobial resistance.
